# Assessing the time intervals between economic recessions

**DOI:** 10.1371/journal.pone.0232615

**Published:** 2020-05-07

**Authors:** Cláudio Tadeu Cristino, Piotr Żebrowski, Matthias Wildemeersch

**Affiliations:** 1 International Institute for Applied Systems Analysis Laxenburg, Laxenburg, Austria; 2 Department of Statistics and Informatics Federal Rural University of Pernambuco Recife, Pernambuco, Brazil; Scuola Superiore Sant’Anna, ITALY

## Abstract

Economic recessions occur with varying duration and intensity and may entail substantial losses in terms of GDP, employment, household income, and investment spending. In this work, we propose a statistical model for the time intervals between recessions that accounts for the state of the economy and the impact of market adjustments and regulatory changes. The model uses a generalized renewal process based on the Gumbel distribution (GuGRP) in which times between consecutive events are conditionally independent. We also present a novel goodness of fit test tailored to the GuGRP that validates the use of the statistical model for the analysis of recessions. Analyzing recessions in the U.S. and Europe, we demonstrate that the statistical model characterizes well recession inter-arrival times and that the model performs better than simpler, commonly used distributions. In addition, the presented statistical model enables us to compare the adjustment processes in different economies and to forecast the occurrence of future recessions.

## Introduction

Recessions are economic contractions following a period of economic expansion. They are part of the business cycle, and do not feature a regular periodicity. Market adjustments and policy measures are capable to revitalize the economy and turn around the trend of economic decline. In the modern economic history of the U.S., policy makers have tried time and time again to address unemployment and declining incomes with recovery measures in the New Deal, the employment act, new institutional frameworks, the Bretton Woods agreement, etc. [1, 2]. In addition to reforms and new regulations, different instruments of fiscal control can be activated, such as adjustments in taxation, public expenditure and debt management to foster economic growth [3]. The efficiency of all these policies can be measured with so-called fiscal multipliers, which behave differently during expansions and recessions [4, 5]. However, these multipliers do not allow us to estimate the time and probability of the next recession.

The lifetime of a system is defined as the time that a system can carry out its function before going into a failure state [6, 7]. In this work, the failure state corresponds with a recession event, and the age of the system is the time that the system has already worked properly, i.e., the time that the economy is in expansion. There are multiple approaches to predict business cycle turning points, such as econometric techniques [8], the spread between short-term and long-term interest rates captured in the yield function [9], or stochastic simulation [10, 11]. Typically, the probability of a recession is determined based on the several indicator variables, such as stock prices, credit market activity, or employment and interest rate [11]. These techniques are valuable, but they do not capture explicitly the effect of system age, system aging, or the effects of policy and market interventions on the state of the economy. There is in fact evidence that the termination probability of recessions is dependent on its duration [12, 13]. This dependence can be captured by different types of survival models, such as the proportional hazard model [14] and the accelerated failure time model [15], which include the effect of covariates in the hazard function. Aforementioned models are however not able to include the effect of interventions following an event on system age. Business cycle durations have typically been modeled using Markov switching processes [16, 17]. Here, we use an alternative approach based on Generalized Renewal Processes (GRP) to model the inter-arrival times of recessions. This method allows us to integrate explicitly the effect of policy interventions and to determine if aging is a real process happening in economic systems.

GRPs integrate simultaneously two sources of stochastic variability in system performance: deterioration through aging and restoration by means of policy interventions. System deterioration can occur through different types of aging processes. No aging indicates that the age of the system has no effect on the residual lifetime. The no-aging property implies a system with no memory, a constant failure rate, and lifetimes distributed according to the exponential distribution. In reliability analysis, positive aging is much more common and means that the expected residual lifetime decreases with age. System restoration deals with repair actions that improve the system after failure. The literature often distinguishes between perfect repair and minimal repair [18]. Perfect repair is applicable, for instance, to engineering systems that consist of a single component that can be replaced, resulting in a system as good as new. Minimal repair requires the replacement of only these components that failed; minimal repair is a reasonable assumption for systems consisting of multiple components all of which have their own failure properties, leaving the system in a state as bad as before the failure. Minimal repair and perfect repair are however specific cases and in realistic settings the degree of restoration can be better described by general repair and the corresponding stochastic process, the GRP. The effectiveness of interventions in general repair can take arbitrary values, and general renewal theory extends the classical renewal theory by including the notion of rejuvenation, indicating the performance of the system after interventions. This idea was firstly presented in [19] and requires to introduce the concept of virtual age, a function that reflects how interventions affect the real age of the system. In the literature, a Weibull-GRP (WGRP) model is usually fitted to observed data in order to infer lifetime probabilities [20–23]. The restoration process is commonly captured by a rejuvenation parameter [22, 24, 25], whereas the deterioration of the system is reflected by the shape and scale parameters of the Weibull distribution regardless of the restoration process [20–22]. The GRP can also be used in an optimization context, where policies are searched for that minimize intervention costs while maintaining certain levels of system performance [24, 26–28].

In reliability analysis and engineering, the Weibull distribution has typically been used to describe the functioning of a serial system that operates well as long as all components operate correctly [29]. In this work, we propose to model the arrival time of recessions by means of a GRP based on the Gumbel distribution. The Gumbel distribution is able to describe a parallel system that stops operating once all components fail, and we use the abstraction of a parallel system as a stylized representation of the economy. The duration of an expansion period is affected by market adjustments taking place during the preceding recession as well as by the policies and reforms introduced to mitigate the causes of this recession. We therefore argue that the periods between consecutive recessions do not constitute a stationary process. We deduce the distribution of the GRP based on the Gumbel distribution, and show how the parameters of the statistical model can be estimated. Importantly, we propose a novel goodness of fit test to demonstrate that the observed recessions of the American and European markets can be represented by the Gumbel-based GRP. We show that the GRP outperforms several stationary distributions, and in addition to the recession incidence rate over time, we also estimate the expected time of the next recession.

## Materials and methods

### Generalized renewal process

**Fig 1:**
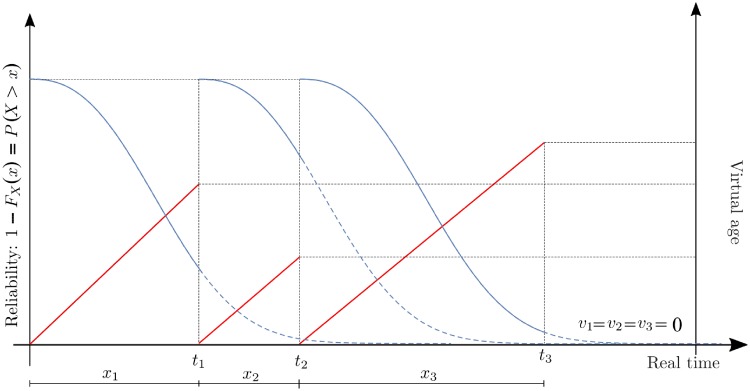
Failure time distribution and effective age. Complementary CDF of the failure time (blue) and effective age (red) for *q* = 0. At the time of recessions, we observe how interventions modify the effective age resulting in a new virtual age *v*_*i*_.

In this work, we model a failure process for an economy starting at time *t* = 0. Each time a failure occurs, represented by a recession, corrections are taking place by means of market adjustments, new regulations, etc., and these corrections bring the economy to a new state different from the state at the onset of the recession. The choice of the initial time and initial conditions is arbitrary, and we allow for interventions that bring the economy into a state better than the initial state of the economy. Let *X*_1_, *X*_2_,… be nonnegative random variables (r.v.’s) that stand for the inter-arrival time of recessions, i.e., the time between the end of a recession and the onset of the next one. To model this failure process, we make use of a general repair model for repairable systems [18]. Specifically, we make use of a generalized renewal process (GRP), where the distribution of the *i*th failure time depends on the partial sum $S_{i-1}=\sum_{j=1}^{i-1}X_{j}$ and the virtual age *v*_*i*−1_ [19]. If the system has the virtual age *v*_*i*−1_ immediately after the (*i* − 1)th recession, then the *i*th failure time *X*_*i*_ follows a GRP with distribution defined as

$$\begin{array}{c} F_{X_{i}}\left(x\;|\;v_{i-1}\right)=P\left[X_{i}\leq x\;|\;V_{i-1}=v_{i-1}\right]=\frac{F_{Y}\left(x+v_{i-1}\right)-F_{Y}\left(v_{i-1}\right)}{1-F_{Y}\left(v_{i-1}\right)}\;, \end{array}$$ (1)

where *F*_*Y*_(⋅) is the failure time distribution of a new system with virtual age *v*_0_ = 0. The notion of virtual age was introduced in [30], and allows to model different degrees of system repair, represented by *q*. In the case of recessions, we will rather refer to system adjustment and the degree of adjustment stands for the corrective changes in the economy following the recession. These corrective actions include fiscal stimuli, regulations of the financial sector, trade reforms, market adjustments, etc. There are two models of adjustment processes with different effects on the system. When the *i*th adjustment only removes damages incurred since the (*i* − 1)th adjustment (virtual age type I), the virtual age can be defined as

$$\begin{array}{c} v_{i}^{\prime }=v_{i-1}^{\prime }+q\cdot x_{i}\;, \end{array}$$ (2)

with q∈R and $v_{0}^{\prime }\geq 0$. When the adjustment affects the damages incurred over the entire lifetime (virtual age type II), the virtual age is defined as

$$\begin{array}{c} v_{i}^{\prime \prime }=q\cdot \left(v_{i-1}^{\prime \prime }+x_{i}\right)\;, \end{array}$$ (3)

with q∈R and $v_{0}^{\prime \prime }\geq 0$. Using recursion, we obtain

$$\begin{array}{c} v_{i}^{\prime }=q\cdot \sum_{j=1}^{i}x_{j} \end{array}$$ (4)

$$\begin{array}{c} v_{i}^{\prime \prime }=\sum_{j=1}^{i}q^{j}\cdot x_{i-j+1}\;, \end{array}$$ (5)

and we observe how the degree of adjustment *q* affects the virtual age differently in the two models. Here, we use a mixture of the both models and we define the virtual age as a convex combination of $v_{i}^{\prime }$ and $v_{i}^{\prime \prime }$ [23]

$$\begin{array}{c} v_{i}=\gamma \cdot \left(v_{i-1}+q\cdot x_{i}\right)+\left(1-\gamma \right)\cdot q\cdot \left(v_{i-1}+x_{i}\right)\;, \end{array}$$ (6)

with *γ* ∈ [0, 1]. Here we assume *q* to be constant. In the special case when *q* = 0, interventions result in perfect repair and failure times follow a renewal process [31], as depicted in Fig 1. If *q* = 1, the interventions are called minimal repair and the virtual age equals the real age of the system. In this case the failure process is a non-homogeneous Poisson process (NHPP) [31]. When 0 < *q* < 1 and with *γ* = 1, the intervention is imperfect and the virtual age acquires an intermediate state [22] between *v*_*i*−1_ and *v*_*i*−1_ + *x*_*i*_. In case *q* > 1, the intervention leads to a condition worse than before intervention. When *q* < 0, interventions result in a system with better than starting conditions. This case can result in a negative virtual age, and requires for distributions with positive support to extend the distribution function over entire R by defining the extended-value distribution function $\tilde{F}\left(\cdot \right)$ as

$$\begin{array}{c} {\tilde{F}}_{X}\left(x\right)=\{\begin{array}{cc} F_{X}\left(x\right) & \text{if}\;\;\;x\in R_{+}\\ 0 & \text{if}\;\;x\in R_{-} \end{array} \end{array}$$ (7)

With abuse of notation, we will use *F*_*X*_(⋅) to denote the extended-value distribution.

**Fig 2:**
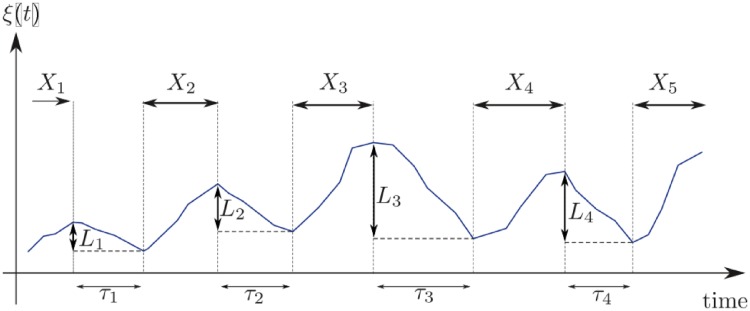
Phases of the business cycle. Trajectory of a representative macroeconomic variable *ξ*(*t*) during expansion and recession episodes.

Unlike temporal point processes that consider events of infinitesimal time, we focus here on events with non-negligible duration. The economy features non-stationary cycles of expansion and contraction, with irregular magnitude and frequency. As a macroeconomic variable *ξ*(*t*) representative for the economy, we use here the GDP (gross domestic product) and follow the trajectory over time reflecting the business cycle (Fig 2). The *i*th recession has duration *τ*_*i*_, while the failure time of the *i*th recession, i.e. the duration of the expansion period, is indicated by *X*_*i*_. The magnitude of the *i*th recession is represented by *L*_*i*_.

Since the duration and magnitude of a recession have an impact on the adjustments in the economy, we modulate in the model the adjustment parameter *q* according to the length and depth of the recession. Therefore, in order to account for the duration of the events as well as the magnitude of the recession, we propose the following types of virtual age

$$\begin{array}{cc} v_{i}^{\prime } & =v_{i-1}^{\prime }+q(1-\exp(-\lambda (\tau_{i},L_{i})))x_{i}\\ v_{i}^{\prime \prime } & =q[v_{i-1}^{\prime \prime }+(1-\exp(-\lambda (\tau_{i},L_{i})))x_{i}]\;, \end{array}$$ (8)

where the factor (1 − exp(−*λ*(*τ*_*i*_, *L*_*i*_))) captures the duration and magnitude of the recession, and the function *λ* is a positive, monotonic function in *τ*_*i*_ and *L*_*i*_. The factor (1 − exp(−*λ*(*τ*_*i*_, *L*_*i*_))) increases with growing values of *τ*_*i*_ and *L*_*i*_. For negative values of *q* (see Results section), this ensures that the effects of interventions are more prominent the longer and deeper the recession becomes. By recursion, we can write

$$\begin{array}{cc} v_{i}^{\prime } & =q\sum_{j=1}^{i}(1-\exp(-\lambda (\tau_{j},L_{j})))x_{j}\\ v_{i}^{\prime \prime } & =\sum_{j=1}^{i}q^{j}(1-\exp(-\lambda (\tau_{i-j+1},L_{i-j+1})))x_{i-j+1}\;. \end{array}$$ (9)

We notice from (9) that the virtual age is determined by the combined effect of *q* and *λ*. By introducing the sensitivity function *λ*, the value of the adjustment parameter *q* is modulated by the duration and depth of each recession. Caution is required for the interpretation of the parameters of the sensitivity function *λ*, since the effect of the modulation depends on the sign of *q*. As introduced in (6), in the remainder of the paper we use a convex combination of the two types of virtual ages, which can be written as follows

$$\begin{array}{c} v_{i}=\gamma \cdot q\cdot \sum_{j=1}^{i}(1-\exp(-\lambda (\tau_{j},L_{j})))x_{j}+\left(1-\gamma \right)\sum_{j=1}^{i}q^{j}(1-\exp(-\lambda (\tau_{i-j+1},L_{i-j+1})))x_{i-j+1}. \end{array}$$ (10)

Here, we will assume that the function *λ*(*τ*_*i*_, *L*_*i*_) is linear in the duration and magnitude of the recession, and we can write

$$\begin{array}{c} \lambda \left(\tau_{i},L_{i}\right)=\delta_{1}\tau_{i}+\delta_{2}L_{i}\;. \end{array}$$ (11)

The following observations about the scope and limitations of the model are in place. First of all, the model is a statistical tool describing the inter-arrival time of recessions, but it does not allow us to draw conclusions about the macroeconomic variables that are used to determine the inter-arrival times. In other words, for values of *q* < 0 the economy reaches a state after expansion better than pre-recession conditions in terms of virtual age, but this does not necessarily imply better than pre-recession GDP levels [32, 33]. Further, due to the limited sample size of the recession data, the adjustment parameter *q* is time-independent and determined for a long period of analysis; *q* can therefore be interpreted as an average level of adjustment. Finally, the limited sample size of our dataset also dictates the functional form of *λ*(*τ*_*i*_, *L*_*i*_), since more complex models cannot be estimated in a statistically meaningful way.

### GRP and the gumbel distribution

The choice of the underlying failure time distribution depends on the considered application. Recessions are typically identified by a decline of GDP during two successive quarters. GDP is an aggregate measure and can be broken down into the contributions of all sectors and industries in an economy. For a single sector, the Weibull and the exponential distribution are both typical candidates for the failure time distribution [34]. Sectoral fluctuations propagate through the economy and results in aggregate outcomes shaping the business cycle. From the perspective of production networks and supply chains, sectoral comovement arises through the linkages in the economy [35]. When the cyclical dynamics are sufficiently persistent and widespread across sectors, then an aggregate business cycle can be identified [36]. This argument justifies why we make here the approximating assumption that during a recession all sectors of the economy are in decline, and motivates the use of a distribution that characterizes the minimum or maximum of a sequence of i.i.d. (independent and identically distributed) random variables [37]. The Gumbel, Fréchet, and Weibull distributions are the only possible limit distributions of the maxima of a sequence of correctly normalized i.i.d. random variables. We can verify that the maxima of a sequence of normalized exponential or Weibull distributed r.v.’s converges to the Gumbel distribution [38]. In other words, the Weibull and exponential distribution belong to the domain of attraction of the Gumbel distribution. We apply these notions now to the case of business cycles, and we can therefore assume that the time that the last sector of the economy goes in decline, corresponding with the onset of a recession, can be described by the Gumbel distribution. Notice that the assumption of a Gumbel distribution is a reasonable assumption in case of severe recessions of global reach. However, since the sectors within an economy are not independent and some sectors can be in expansion during a recession, modeling inter-arrival times of recessions based on the Gumbel distribution has to be understood as a useful approximation. The approximation can be further refined by using order statistics different from the largest order statistic at the expense of tractability.

The cumulative distribution function (CDF) of the Gumbel distribution with location parameter α∈R and scale parameter *β* > 0 is given by

$$\begin{array}{c} F_{Y}\left(y;\alpha ,\beta \right)=1-\exp(-\exp(\frac{y-\alpha }{\beta }))\;, \end{array}$$ (12)

with corresponding probability density function (PDF)

$$\begin{array}{c} f_{Y}\left(y;\alpha ,\beta \right)=\frac{1}{\beta }\exp(-\frac{y-\alpha }{\beta }-\exp(\frac{y-\alpha }{\beta }))\;. \end{array}$$ (13)

The hazard function for the Gumbel distribution is:

$$\begin{array}{c} h_{Y}\left(y;\alpha ,\beta \right)=\frac{1}{\beta }\exp(\frac{y-\alpha }{\beta }). \end{array}$$ (14)

If *Y* ∼ Gumbel(*α*, *β*), the expected value and variance can be expressed as $E\left[Y\right]=\alpha +c_{0}\beta$ and $\text{Var}\left[Y\right]=\frac{1}{6}\pi^{2}\beta^{2}$, where *c*_0_ is the Euler-Mascheroni constant [39].

Let {*X*_*n*_}_*n*≥1_ be a sequence of failure times following a Gumbel-GRP (GuGRP) process. Using (1) and (12) the distribution of the failure time *X*_*i*_ following the GuGRP can be written as

$$\begin{array}{ccc} F_{X_{i}}\left(x\;|\;v_{i-1};\alpha ,\beta \right) & = & \frac{1-\exp(-\exp(\frac{x+v_{i-1}-\alpha }{\beta }))-(1-\exp(-\exp(\frac{v_{i-1}-\alpha }{\beta })))}{1-(1-\exp(-\exp(\frac{v_{i-1}-\alpha }{\beta })))}\\ & = & 1-\exp(\exp(\frac{v_{i-1}-\alpha }{\beta })-\exp(\frac{x-\alpha +v_{i-1}}{\beta }))\;, \end{array}$$ (15)

with corresponding PDF

$$\begin{array}{c} f_{X_{i}}\left(x\;|\;v_{i-1};\alpha ,\beta \right)=\frac{1}{\beta }\;\exp(\frac{x-\alpha +v_{i-1}}{\beta }-\exp(\frac{x-\alpha +v_{i-1}}{\beta })+\exp(\frac{-\alpha +v_{i-1}}{\beta }))\;, \end{array}$$ (16)

and hazard function

$$\begin{array}{c} h_{X_{i}}\left(x\;|\;v_{i-1};\alpha ,\beta \right)=\frac{1}{\beta }\exp(\frac{x-\alpha +v_{i-1}}{\beta })\;. \end{array}$$ (17)

**Fig 3:**
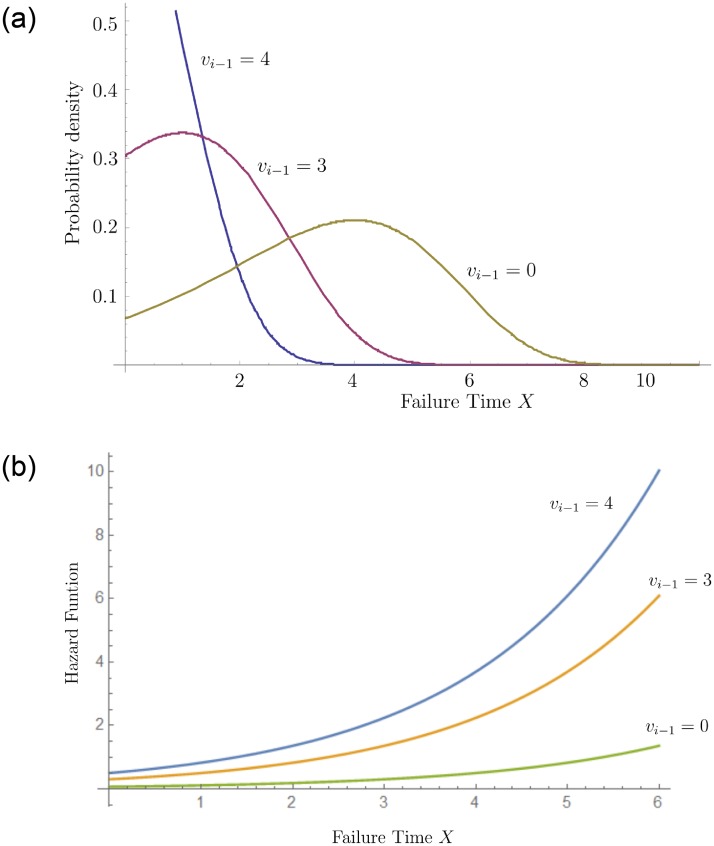
GuGRP distribution and hazard function. GuGRP PDF for different values of the virtual age. (*α* = 4 and *β* = 2) (L). Hazard function for different values of the virtual age (*α* = 4 and *β* = 2) (R).

The effect of virtual age on the probability density of a recession can be seen in Fig 3a, where we observe that increasing virtual age reduces the remaining system lifetime. The effect of the virtual age on the hazard function is shown in Fig 3b, where we observe the positive ageing property and that the slope of the hazard function increases with virtual age.

### Parameter estimation

In order to estimate the parameters of the statistical model, we make use of maximum likelihood estimates (MLE). Due to the limited sample size of recession data, we will assume at first instance that the virtual age is affected by the recession duration, but not by the depth of the recession, and therefore we have *λ*(*τ*_*i*_) = *δτ*_*i*_. The vector of parameters to be estimated is given by $\underline{\theta }=\left(\alpha ,\beta ,\gamma ,\delta ,q\right)$. Considering the conditional independence of the failure times *X*_*i*_, the likelihood function of $\underline{\theta }$ can be expressed as follows

$$\begin{array}{cc} L(\underline{\theta }\;|\;x_{1},\ldots ,x_{n}) & =\prod_{j=1}^{n}f_{T_{j}}\left(x_{j}\;|\;v_{j-1};\alpha ,\beta ,\gamma ,\delta ,q\right)\\ & =\prod_{j=1}^{n}\frac{1}{\beta }\exp(\frac{x_{j}-\alpha +v_{j-1}}{\beta }-\exp(\frac{x_{j}-\alpha +v_{j-1}}{\beta })+\exp(\frac{-\alpha +v_{j-1}}{\beta }))\;, \end{array}$$ (18)

with *v*_*j*−1_ given by (10) and *v*_0_ = 0. Consequently, the log-likelihood of $\underline{\theta }$ can be written as

$$\begin{array}{c} \ell \left(\underline{\theta }\;|\;x_{1},\ldots ,x_{n}\right)=\log[L(\underline{\theta }\;|\;x_{1},\ldots ,x_{n})]\\ =-n\log\left(\beta \right)+\sum_{j=1}^{n}(\frac{x_{j}-\alpha +v_{j-1}}{\beta }-\exp(\frac{x_{j}-\alpha +v_{j-1}}{\beta })+\exp(\frac{-\alpha +v_{j-1}}{\beta })). \end{array}$$

The MLE for $\underline{\theta }$ is found by solving the following system of equations

$$\begin{array}{c} \left\{ \begin{array}{ccc} \frac{\partial \ell }{\partial \alpha }=0; & \frac{\partial \ell }{\partial \beta }=0; & \frac{\partial \ell }{\partial \delta }=0;\\ \frac{\partial \ell }{\partial \gamma }=0; & \frac{\partial \ell }{\partial q}=0\;, \end{array}\right. \end{array}$$ (19)

which can be written explicitly as

$$\begin{array}{c} \left\{ \begin{array}{c} \sum_{j=1}^{n}e^{-\frac{\alpha }{\beta }}[e^{\frac{x_{j}+\gamma qs_{j-i}+\left(1-\gamma \right)\sum_{r=0}^{j-1}q^{r}y_{j-r}}{\beta }}-e^{\frac{\gamma qs_{j-i}+\left(1-\gamma \right)\sum_{r=0}^{j-1}q^{r}y_{j-r}}{\beta }}]=n\\ \sum_{j=1}^{n}\frac{x_{j}-\alpha +\gamma qs_{j-i}+\left(1-\gamma \right)\sum_{r=0}^{j-1}q^{r}y_{j-r}}{\beta }[e^{\frac{x_{j}-\alpha +\gamma qs_{j-i}+\left(1-\gamma \right)\sum_{r=0}^{j-1}q^{r}y_{j-r}}{\beta }}-1]\\ \;-\frac{-\alpha +\gamma qs_{j-i}+\left(1-\gamma \right)\sum_{r=0}^{j-1}q^{r}y_{j-r}}{\beta }\cdot e^{\frac{-\alpha +\gamma qs_{j-i}+\left(1-\gamma \right)\sum_{r=0}^{j-1}q^{r}y_{j-r}}{\beta }}=n\\ \sum_{j=1}^{n}\frac{qs_{j-1}-\sum_{r=0}^{j-1}q^{r}y_{j-1}}{\beta }(1-e^{\frac{x_{j}-\alpha +\gamma qs_{j-i}+\left(1-\gamma \right)\sum_{r=0}^{j-1}q^{r}y_{j-r}}{\beta }}+e^{\frac{-\alpha +\gamma qs_{j-i}+\left(1-\gamma \right)\sum_{r=0}^{j-1}q^{r}y_{j-r}}{\beta }})\\ =0\\ \sum_{j=1}^{n}\frac{\gamma s_{j-1}+\left(1-\gamma \right)\sum_{r=0}^{j-1}rq^{r-1}y_{j-1}}{\beta }\\ (1-e^{\frac{x_{j}-\alpha +\gamma qs_{j-i}+\left(1-\gamma \right)\sum_{r=0}^{j-1}q^{r}y_{j-r}}{\beta }}+e^{\frac{-\alpha +\gamma qs_{j-i}+\left(1-\gamma \right)\sum_{r=0}^{j-1}q^{r}y_{j-r}}{\beta }})=0\\ \sum_{j=1}^{n}\sum_{r=0}^{j-1}\tau_{r}\exp(-\delta \tau_{r})x_{r}\{1-\exp(\frac{x_{j}-\alpha +qy_{r}}{\beta })+\exp(\frac{-\alpha +qy_{r}}{\beta })\}=0 \end{array}\right. \end{array}$$ (20)

with $s_{k}=\sum_{r=1}^{k}\left(1-\exp\left(-\lambda \left(\tau_{r},L_{r}\right)\right)\right)x_{r}$ and *y*_*k*_ = (1 − exp(−*λ*(*τ*_*k*_, *L*_*k*_)))*x*_*k*_.

### Goodness of fit test for the GuGRP

As validation of the GuGRP model, we need to evaluate how good the model fits the observations. In this section, we develop a goodness of fit test (GoFT) for the GuGRP based on a transformation of the failure time and virtual age into an exponential distribution. This idea has been used in the literature to construct a GoFT for the Weibull distribution [40]. After the transformation, a GoFT for the exponential distribution can be applied. In the following proposition, we establish the relationship between the exponential distribution and the sum of failure time and virtual age.

**Proposition 1** Let (*W*_1_, …, *W*_*n*_), *n* ≥ 1, be a random vector such that

$$\begin{array}{c} W_{i}=\exp(\frac{X_{i}+V_{i-1}-\alpha }{\beta })-\exp(\frac{V_{i-1}-\alpha }{\beta })\;, \end{array}$$ (21)

*where X*_*i*_ *conditioned on the virtual age V*_*i*−1_ *follows a GuGRP with parameters α and β*. *Then the following claims hold true*

*(i) W*_*i*_ *follows an exponential distribution with parameter 1*,
*(ii) W*_*i*_, …, *W*_*n*_ *are identically distributed and mutually independent*.

**Proof 1** *See* S1 Dataset.

We now provide an algorithm that applies Proposition 1 to create a simple GoFT. We will use this algorithm in the results section to demonstrate that the inter-arrival times of recessions can be described by the GuGRP.

**Algorithm 1:** Gumbel generalized renewal process GoFT.

**input**: (*x_i_*, *ρ_i_*, *L_i_*)_*i*=1,..*n*_ for each event; the significance level for the GuGRP goodness of fit test, ζ

**output**: The conclusion of the GuGRP goodness of fit test

**begin**

  GugrpFitTest((*x*_*i*_, *τ*_*i*_, *L*_*i*_)_*i*=1..*n*_):

  /* Selection of the GuGRP model based on the MLE using observations (*x*_1_, …, *x*_*n*_)     */

   $\left(\hat{\alpha },\hat{\beta },\hat{\gamma },\hat{\delta },\hat{q}\right)\leftarrow \text{MLE}\left(\left(x_{i}\right),\left(\tau_{i}\right)\right)$

  /* Transformation of failure time and virtual age yielding **w** = (*w*_1_, …, *w*_*n*_)         */

  *v*_0_ = 0

  **for** *i* ← 1 **to** *n* **do**

  $v_{i}=\hat{\gamma }\cdot \hat{q}\sum_{j=1}^{i}(1-\exp(-\hat{\delta }\tau_{j}))x_{j}+\left(1-\hat{\gamma }\right)\sum_{j=1}^{i}{\hat{q}}^{i-j+1}(1-\exp(-\hat{\delta }\tau_{j}))x_{j}$. // See Eq (10)

  $w_{i}=\exp(\frac{x_{i}+v_{i-1}-\hat{\alpha }}{\hat{\beta }})-\exp(\frac{v_{i-1}-\hat{\alpha }}{\hat{\beta }})$ // See Proposition (1)

  /* Calculation of the p-value p* of **w** corresponding to the exponential distribution       */

  $p^{*}\leftarrow \text{ExponentialPValue}\left(w,1\right)$ The significance level of **w** for the Exponential distribution with mean 1

  **if** p* ≥ *ζ* **then**

   **Do not reject** *H*_0_: There is no evidence that *x*_1_, …, *x*_*n*_ do not follow the GuGRP with parameters $\left(\hat{\alpha },\hat{\beta },\hat{\gamma },\hat{\delta },\hat{q}\right)$

 **else**

  **Reject** *H*_0_: There is evidence that *x*_1_, …, *x*_*n*_ do not follow the GuGRP with parameters $\left(\hat{\alpha },\hat{\beta },\hat{\gamma },\hat{\delta },\hat{q}\right)$

## Results and discussion

This section provides results on several domains. First of all, we validate the GuGRP and benchmark the performance of the GuGRP with respect to the exponential distribution and the Gumbel distribution, demonstrating that the GuGRP outperforms both other distributions. We also provide some results on the predictive power of the statistical method. We will make use of two datasets on American (S1 Table) and European recessions (S2 Table). The dataset on American recessions is split in two subsets, splitting the data before and after the Great Depression [11, 41, 42]. This division is based on the argument that the behavior of the economy is substantially different before and after the Great Depression. Due to the limited sample size of the observations, we assume a constant degree of adjustment *q* in both periods.

### GuGRP captures well the succession of recessions

Using an MLE procedure, we obtain numerically the parameters of the GuGRP for American recessions as given in Table 1. For the periods before and after the Great Depression, we used the GoFT defined in algorithm 1. A Kolmogorov-Smirnov test is performed on the transformed exponential r.v.’s. Before the Great Depression, the null hypothesis stating that the transformed data is distributed according to the exponential distribution with rate parameter *μ* = 1, is not rejected at the 5% level (*p*-value = 0.758). Similarly, after the Great Depression the null hypothesis is also not rejected at the 5% level (*p*-value = 0.836). Other hypothesis tests are presented in Table 1 with similar results. The hypothesis tests demonstrate that the GuGRP is an appropriate model to represent the inter-arrival time of recessions, while accounting for post-recession adjustment effects due to regulations, reforms, stimulus packages or market adjustments. Our results indicate that adjustments of economic systems encapsulated in the virtual age are detectable in the data. We notice however important differences between the parameters before and after the Great Depression. First, before the Great Depression the behavior of the virtual age is well described by the type II model since *γ* ≈ 0, and interventions affect damages incurred over the entire lifetime. After the Great Depression, the virtual age is a mixture of models type I and type II. Another important difference is that the degree of adjustment *q* improves considerably after the Great Depression. In fact, after the Great Depression *q* is negative, indicating that after a recession the economy is in a better state than before the onset of the recession.

**Table 1:** Model parameters for American recessions. MLE and GoFT for GuGRP parameters of American recessions.

Parameter estimates	$\hat{\alpha }$	$\hat{\beta }$	$\hat{\gamma }$	$\hat{\delta }$	$\hat{q}$
Before Great Depression	4.13292	0.788003	0.0636201	21.3288	0.395581
After Great Depression	3.49627	3.01529	0.403901	0.249004	−0.874915
	before Great Depression	after Great Depression			
test *H*_0_: data follows GuGRP	Statistic	*p*-value	Statistic	*p*-value	
Anderson-Darling	0.493742	0.750803	0.340753	0.90305	
Cramér-von Mises	0.0688818	0.758445	0.0512278	0.868788	
Pearson *χ*^2^	3.8	0.70372	5.92308	0.313776	

**Fig 4:**
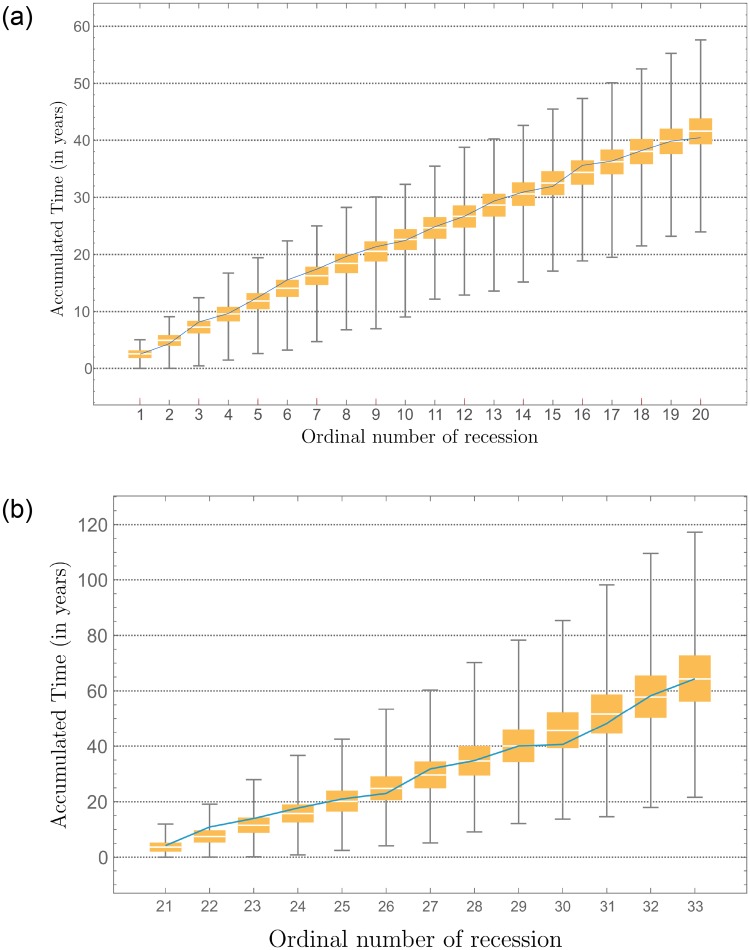
Simulated recession times. Times of recessions before the Great Depression (a) and after the Great Depression (b). The blue line represents the real data (NBER, 2016) and the box plots are representations of simulated data using the estimated GuGRP model. The vertical axis represents time in years.

Another approach for model validation is by simulating the GuGRP model and comparing it with real data. In order to do so, we need to generate both inter-recession times and recession durations. We generate samples of inter-recession times *X*_*i*_ by means of inverse transform sampling [43], where *X*_*i*_ can be generated using the inverse of the CDF given by

$$\begin{array}{c} x_{i}=\beta \log(\exp(\frac{-\alpha +v_{i-1}}{\beta })-\log\left(1-u\right))+\alpha -v_{i-1},\;\;i=1,\ldots ,m\;, \end{array}$$ (22)

with *u* uniform r.v.’s in [0, 1]. The recession duration is modeled as a Weibull independent r.v., and the null hypothesis that the data is distributed according to the Weibull distribution with shape parameter *α* = 0.845723, scale parameter *β* = 0.891526, and location parameter *μ* = 0.591218 is not rejected at the 5% level based on the Cramér-von Mises test (*p*-value 0.712) [44]. The duration of recessions has also been modeled by means of an exponential distribution [45], which is a special case of the Weibull distribution. We generated 100 000 samples of size 20 (number of recessions before the Great Depression), and the results are shown in Fig 4. The simulation results visualize that cycles of recessions and expansions are captured well by the GuGRP for inter-recession times and the Weibull distribution for the recession duration.

The depth of a recession modifies the measures that will be taken in response to the economic decline. The sequence of U.S. recession depths from the second quarter of 1947 until the third quarter of 2017 can be found in S3 Table. We now include the depth of the recession in the function *λ*(*τ*_*i*_, *L*_*i*_) = *δ*_1_ *τ*_*i*_ + *δ*_2_ *L*_*i*_, and verify the effects on the quality of the statistical model. Using MLE, we numerically find the estimates of the model parameters as given in Table 2. Using the GoFT based on Proposition 1, the null hypothesis that the transformed data is distributed according to the exponential distribution with parameter *μ* = 1 is not rejected at the 5% level based on the Kolmogorov-Smirnov test (*p*-value is 0.879).

**Table 2:** Model parameters for American recessions. MLE for the parameters of GuGRP considering the recession duration and depth.

Recessions times	$\hat{\alpha }$	$\hat{\beta }$	$\hat{\gamma }$	$\hat{\delta_{1}}$	$\hat{\delta_{2}}$	$\hat{q}$
Nov/1948-Dec/2007	2.59534	3.48206	0.999723	5.52717	4.06923	−0.119704

**Fig 5:**
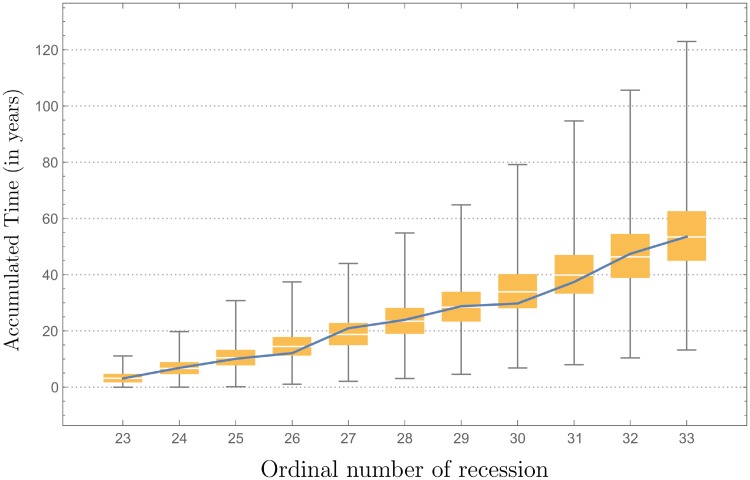
Simulated recession times. Last 11 U.S. recessions based on GuGRP model considering the depth of the recessions. The vertical axis represents time in years.

We generated 100 000 samples with size 11 (number of recessions after the Great Depression) based on the GuGRP model with parameters defined in Table 2, and we depict the sequence of successive recessions in Fig 5. As a result of including the recession depth in the model, the accuracy of the statistical model has improved in terms of expected values.

We now test the GuGRP for the last 12 European recessions, for which the data can be found in Table 4 of S2 Table. In case of virtual age type I, the parameters can be estimated numerically as $\hat{\alpha }=2.6113$, $\hat{\beta }=1.33585$, *γ* = 1, $\hat{\delta }=36.8596$, and $\hat{q}=-0.0109307$. We apply the GoFT described in algorithm 1, and perform the Kolmogorov-Smirnov test. The null hypothesis that the transformed data is distributed according to the exponential distribution with parameter *μ* = 1 is not reject at the 5% level with *p*-value = 0.727. As for the American recessions after the Great Depression, we notice that the degree of adjustment *q* is negative, indicating that the economy achieves a better state through corrective measures and adjustment processes taking place after each recession. However, this effect is more pronounced for American recessions, which can imply that historically the American economy has been more effective in their adjustments than the European economy. Since the European Union does not constitute a fiscal union, it is in line with the expectations that the response to recessions is less effective in Europe. However, the American and European markets are not isolated and interact with each other, and therefore their business cycles are correlated [46]. Still, the difference in efficiency of adjustment processes can be perceived in our results.

### GuGRP outperforms models that do not include the quality of adjustment processes

In order to demonstrate the necessity of including the quality of adjustment processes into the failure time distribution, we test if it is possible to fit the recession data to simpler stochastic processes, such as the exponential distribution and the Gumbel renewal process.

First, we verify if recessions can be represented by a homogeneous Poisson process, with independent and exponentially distributed times between recessions. We perform a Kolmogorov-Smirnov test, and the null hypothesis that the data is distributed according to the exponential distribution with parameter *μ*^−1^ = 1/2.11 yr^−1^ is rejected at the 5% level with *p*-value equal to 0.00153424. In other words, it is not possible to represent recessions by means of a homogeneous Poisson process.

**Fig 6:**
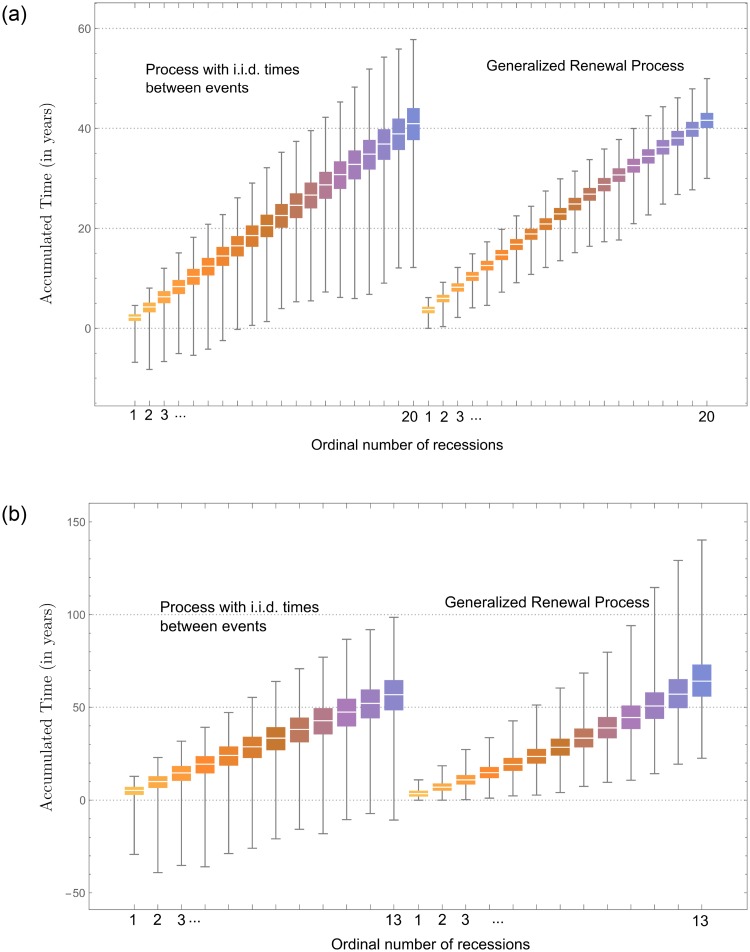
Value of including adjustment processes in statistical model. Recession times before the Great Depression (a) and after the Great Depression (b). The left side in each subplot represents a Gumbel renewal process, whereas the right side represents the GuGRP. The box plots visualize simulated data, and the vertical axis represents time in years.

When we model the recession process according to a Gumbel renewal process, we also notice significant differences with respect to the GuGRP, as depicted in Fig 6. From the figure, we observe that the Gumbel renewal process allows for negative failure times, which is not meaningful. Moreover, the variance of the Gumbel renewal process is considerably larger than the variance of the GuGRP. As to the expected failure time, we notice a considerable difference with respect to the GuGRP, for which the expected failure time corresponds well with the observed data. In conclusion, the dependence between consecutive recessions reflected in the generalized renewal process by means of the virtual age cannot be captured by simpler processes of independently distributed r.v.’s.

### Predictive power of GuGRP

**Fig 7:**
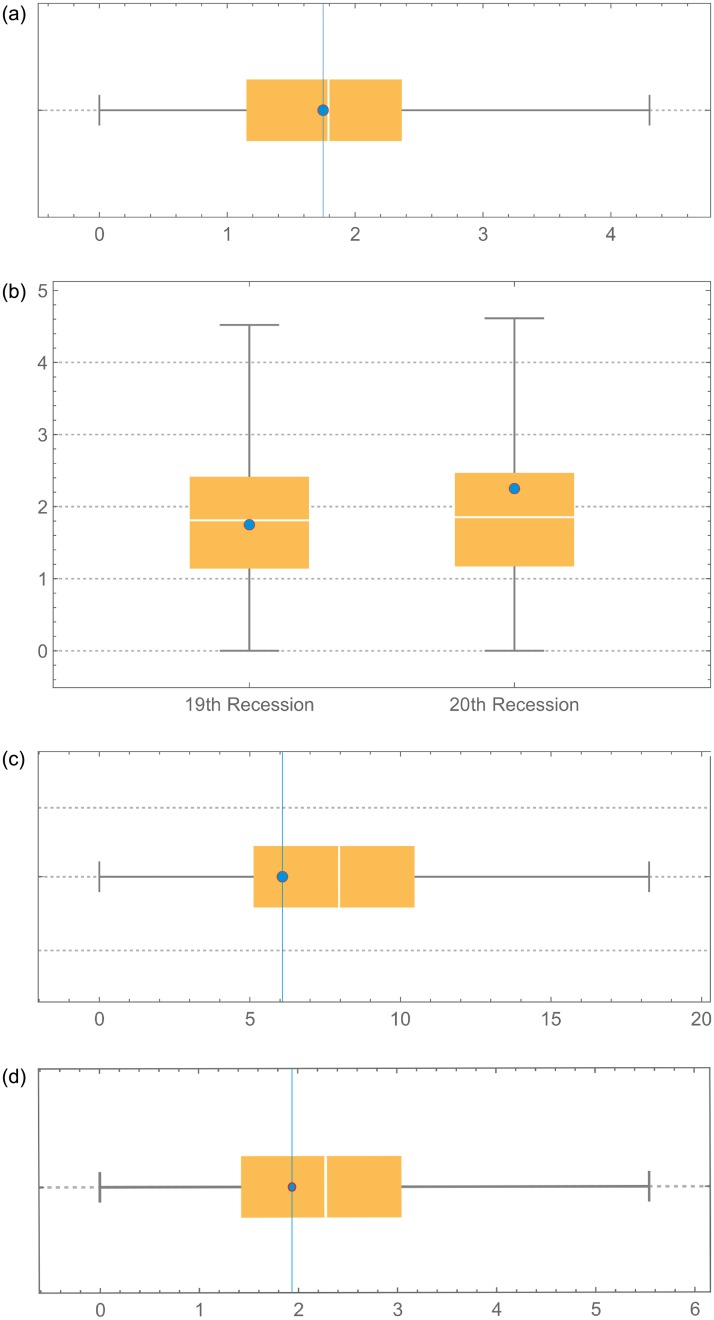
Predictive power of GuGRP model. Blue points indicate the real value of the failure time, and horizontal axis represents time in years. (a) box plot for prediction of 20th U.S. recession. (b) box plot for prediction of U.S. recessions 19 and 20. (c) box plot for prediction of U.S. recession in December 2007 based on 8 previous recessions. (d) box plot of 12th European recession based on previous 11 recessions.

We assess the predictive power of the GuGRP model by means of different out-of-sample predictions. For that purpose, we use the first 19 U.S. recessions before the Great Depression and predict the next recession. The model parameters are estimated numerically and MLE yields $\hat{\alpha }=2.78549$, $\hat{\beta }=0.942578$, $\hat{\gamma }\approx 1$, $\hat{\delta }=20.6964$, and $\hat{q}=0.0188325$. These parameters are used to simulate the next recession (100 000 runs) and from the results in Fig 7a we find that the expected failure time corresponds very well with the real failure time. Using the first 18 U.S. recessions, we find by MLE the parameters $\hat{\alpha }=2.77702$, $\hat{\beta }=0.982942$, $\hat{\gamma }\approx 1$, $\hat{\delta }=20.122$, and $\hat{q}=0.0202115$. We use these parameters to predict out-of-sample the next two recessions (Fig 7b). Also in this case, we observe that there is good agreement between the expected and real failure times. If we use the first subset of U.S. recessions to predict the first recession of the second subset, the expected value of the next recession ${\bar{X}}_{21}=1,71864\;\text{yr}$ with standard deviation equal to 0.8101 yr does not correspond well with the real value *X*_21_ = 4.169863 yr. This result indicates extraordinary changes in the economy following the Great Depression, which were not observed before the Great Depression. In the period after the Great Depression, we use 8 recessions between August 1957 and March 2001 to predict the recession in December 2007, according to [42]. Using MLE, we find the parameters $\hat{\alpha }=2.92227$, $\hat{\beta }=4.04123$, $\hat{\gamma }\approx 1$, $\hat{\delta }=29.4753$, and $\hat{q}=-0.145639$. We notice that the degree of adjustment *q* is considerably lower after the Great Depression, which points to more effective adjustments in response to recessions after the Great Depression. The prediction results are shown in Fig 7c and show worse predictive power compared to the period before the Great Depression both in terms of expected value and standard deviation, mainly due to the small size of the available sample. For the European recessions, we use the 11 recessions between 1963 and 2008 to predict the 2011 recession. MLE yields the parameters $\hat{\alpha }=2.53209$, $\hat{\beta }=1.28065$, $\hat{\gamma }=0.999984$, $\hat{\delta }=16.7434$, and $\hat{q}=-0.0262036$. We observe from the simulation results in Fig 7d that the GuGRP applied to European recessions also features good predictive power.

In the out-of-sample predictions of this section, the MLE procedure consistently provides parameter values corresponding with virtual age type I (*γ* ≈ 1). In case of small sample size it is advisable to use virtual age type I since this reduces the number of parameters to be estimated. Moreover, virtual age type I provides a more intuitive interpretation, and complications are avoided that appear with virtual age type II in case of negative values of the adjustment parameter *q*.

**Fig 8:**
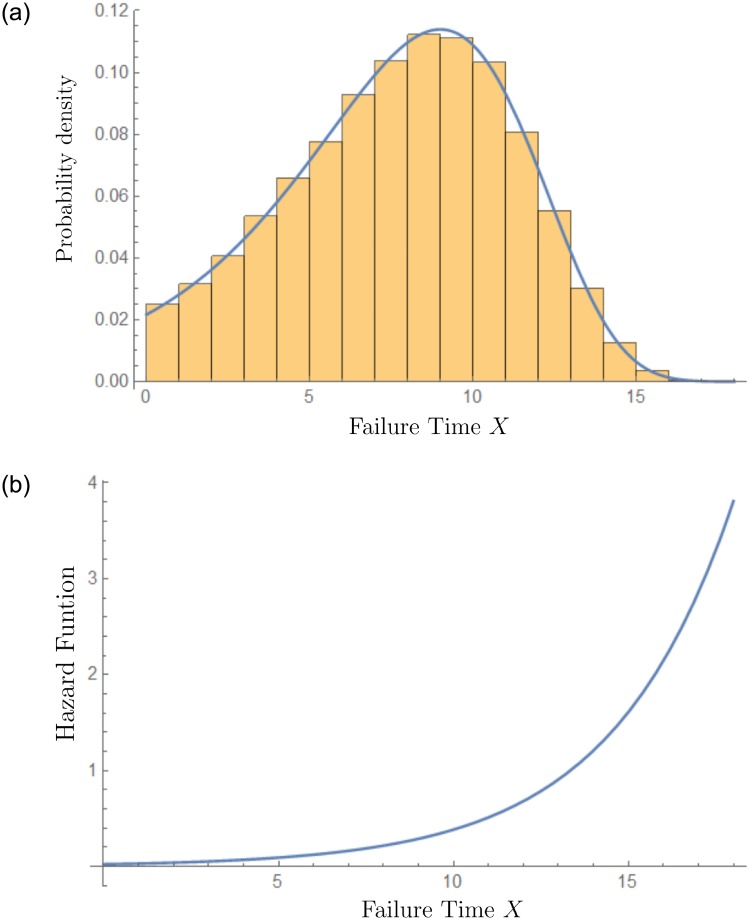
Prediction of next U.S. recession. (a) PDF of the failure time. (b) Hazard function of the failure time distribution.

The former analysis gives us a quantitative sense of the predictive power of the GuGRP applied to recessions. An important application of the statistical method is to forecast next recessions. In the case of the U.S., we use data from the last 11 recessions and obtain the parameters $\hat{\alpha }=2.59534$, $\hat{\beta }=3.48206$, $\hat{\gamma }=0.999723$, ${\hat{\delta }}_{1}=5.52717$, ${\hat{\delta }}_{2}=4.06923$, and $\hat{q}=-0.119704$. In Fig 8, we can observe the PDF and the hazard function of the next U.S. recession based on 100 000 simulations. The expected value of the failure time is ${\bar{X}}_{34}=7.8186\;\text{yr}$ with standard deviation 3.34734 yr.

## Conclusions

In this work, we proposed a statistical model to describe the time intervals between economic recessions by means of a generalized renewal process. This model includes multiple components that are essential in the study of economic recessions. In particular, the model captures the combined effect of adjustments in the economy and policy interventions on the remaining time before the next recession. Moreover, as we are considering events with non-negligible duration, we also incorporate recession duration and recession depth in the effectiveness of interventions. Owing to the aggregate nature of metrics that describe the state of an economy, a distribution that represents the maximum of a sequence of i.i.d. random variables is appropriate, and the failure time distribution and hazard function are derived according to the GuGRP. Importantly, we proposed a goodness-of-fit test based on the transformation of failure times, and we evaluated the suitability of the GuGRP in the specific application of economic recessions. We applied the statistical model to recent recessions in the U.S. and European markets, and our results show that the GuGRP features good descriptive and predictive power. Moreover, we demonstrated that it is not possible to represent recession data with models that do not include the degree of adjustment and ignore the dependence structure of the failure times. Through the degree of adjustment, the statistical model allows us to compare the efficiency of adjustments in different markets. Our results reflect that adjustments in the American economy were more profound than in Europe after the Great Depression. Finally, the statistical model can be applied to inform policy makers of the estimated onset of the next recession.

## Supporting information

S1 Dataset Proof of Proposition 1. (PDF)

S1 Table U.S. recessions. National Bureau of Economic Research—NBER data.
(PDF)

S2 Table European recessions [47]. (PDF)

S3 Table Depth of U.S. recessions [48]. (PDF)

## References

[1] Rosenberg S. American economic development since 1945: Growth, decline and rejuvenation. Palgrave Macmillan; 2002.

[2] Hawley EW. The New Deal and the problem of monopoly. Princeton University Press; 2015.

[3] Arrow KJ, Kruz M. Public investment, the rate of return, and optimal fiscal policy. RFF Press; 2013.

[4] Blinder AS, Solow RM, et al Does fiscal policy matter? vol. 144 Econometric Research Program, Princeton University; 1972.

[5] Auerbach AJ, Gorodnichenko Y. Fiscal multipliers in recession and expansion In: Fiscal policy after the financial crisis. University of Chicago Press; 2012 p. 63–98.

[6] Deshpande JV, Kochar SC, Singh H. Aspects of positive ageing. Journal of Applied Probability. 1986;23(3):748–758. 10.2307/3214012

[7] Lai CD, Xie M. Concepts and Applications of Stochastic Ageing. Stochastic Ageing and Dependence for Reliability. 2006; p. 7–70.

[8] Watson MW. Using econometric models to predict recessions. Economic Perspectives. 1991;(Nov):14–25.

[9] Rudebusch GD, Williams JC. Forecasting recessions: The puzzle of the enduring power of the yield curve. Journal of Business & Economic Statistics. 2009;27(4):492–503. 10.1198/jbes.2009.07213

[10] Stock JH, Watson MW. Business cycles, indicators, and forecasting. vol. 28 University of Chicago Press; 2008.

[11] Chauvet M. An econometric characterization of business cycle dynamics with factor structure and regime switching. International economic review. 1998; p. 969–996. 10.2307/2527348

[12] Sichel DE. Business cycle duration dependence: A parametric approach. The Review of Economics and Statistics. 1991; p. 254–260. 10.2307/2109515

[13] Diebold FX, Rudebusch GD. A nonparametric investigation of duration dependence in the American business cycle. Journal of political Economy. 1990;98(3):596–616. 10.1086/261696

[14] Elbers C, Ridder G. True and spurious duration dependence: The identifiability of the proportional hazard model. The Review of Economic Studies. 1982;49(3):403–409. 10.2307/2297364

[15] Robins J, Tsiatis AA. Semiparametric estimation of an accelerated failure time model with time-dependent covariates. Biometrika. 1992;79(2):311–319.

[16] Kim CJ, Nelson CR. Has the US economy become more stable? A Bayesian approach based on a Markov-switching model of the business cycle. Review of Economics and Statistics. 1999;81(4):608–616. 10.1162/003465399558472

[17] Filardo AJ, Gordon SF. Business cycle durations. Journal of econometrics. 1998;85(1):99–123. 10.1016/S0304-4076(97)00096-1

[18] Kijima M. Some Results for Repairable Systems with General Repair. Journal of Applied Probability. 1989;26(1):89–102. 10.2307/3214319

[19] Kijima M, Sumita U. A useful generalization of renewal theory: counting processes governed by non-negative Markovian increments. Journal of Applied Probability. 1986;23(1):71–88. 10.1017/S002190020010628X

[20] Jiménez P, Villalón R. Generalized renewal process as an adaptive probabilistic model. In: Transmission & Distribution Conference and Exposition: Latin America, 2006. TDC’06. IEEE/PES. IEEE; 2006. p. 1–6.

[21] Veber B, Nagode M, Fajdiga M. Generalized renewal process for repairable systems based on finite Weibull mixture. Reliability Engineering & System Safety. 2008;93(10):1461–1472. 10.1016/j.ress.2007.10.003

[22] Yañez M, Joglar F, Modarres M. Generalized renewal process for analysis of repairable systems with limited failure experience. Reliability Engineering & System Safety. 2002;77(2):167–180. 10.1016/S0951-8320(02)00044-3

[23] Ferreira RJ, Firmino PRA, Cristino CT. A mixed kijima model using the weibull-based generalized renewal processes. PloS one. 2015;10(7):e0133772 10.1371/journal.pone.0133772 26197222 PMC4511187

[24] Makis V, Jardine AKS. A note on optimal replacement policy under general repair. European Journal of Operational Research. 1993;69(1):75–82. 10.1016/0377-2217(93)90092-2

[25] Lugtigheid D, Banjevic D, Jardine AK. System repairs: When to perform and what to do? Reliability Engineering & System Safety. 2008;93(4):604–615.

[26] Krivtsov V. A Monte Carlo Approach to Modeling and Estimation of the Generalized Renewal Process in Repairable System Reliability Analysis. University of Maryland, College Park; 2000.

[27] Love CE, Zhang ZG, Zitron MA, Guo R. A discrete semi-Markov decision model to determine the optimal repair/replacement policy under general repairs. European Journal of Operational Research. 2000;125(2):398–409. 10.1016/S0377-2217(99)00009-0

[28] Scarsini M, Shaked M. On the value of an item subject to general repair or maintenance. European Journal of Operational Research. 2000;122(3):625–637. 10.1016/S0377-2217(99)00078-8

[29] Rinne H. The Weibull distribution: A handbook. Chapman and Hall/CRC; 2008.

[30] Kijima M, Morimura H, Suzuki Y. Periodical replacement problem without assuming minimal repair. European Journal of Operational Research. 1988;37(2):194–203. 10.1016/0377-2217(88)90329-3

[31] Modarres M, Kaminskiz M, Krivstov V. Realiability Engineering and Risk Analysis: A Practical Guide Plastics Engineering. Marcel Dekker Incorporated; 1999 Available from: http://books.google.com.br/books?id=IZ5VKc-Y4_4C.

[32] Ball L. Long-term damage from the Great Recession in OECD countries. European Journal of Economics and Economic Policies: Intervention. 2014;11(2):149–160.

[33] Dosi G, Pereira MC, Roventini A, Virgillito ME. Causes and consequences of hysteresis: aggregate demand, productivity, and employment. Industrial and Corporate Change. 2018;27(6):1015–1044. 10.1093/icc/dty010

[34] Rausand M, Høyland A. System reliability theory: models, statistical methods, and applications. vol. 396 John Wiley & Sons; 2003.

[35] Carvalho VM. From micro to macro via production networks. Journal of Economic Perspectives. 2014;28(4):23–48. 10.1257/jep.28.4.23

[36] Stock JH, Watson MW. Business cycle fluctuations in US macroeconomic time series. National Bureau of Economic Research; 1998.

[37] Embrechts P, Klüppelberg C, Mikosch T. Modelling extremal events: for insurance and finance. vol. 33 Springer Science & Business Media; 2013.

[38] Resnick SI. Extreme values, regular variation and point processes. Springer; 2013.

[39] Conway JH, Guy R. The book of numbers. Springer Science & Business Media; 2012.

[40] de Oliveira CCF, Cristino CT, Firmino PRA. In the kernel of modelling repairable systems: a goodness of fit test for Weibull-based generalized renewal processes. Journal of Cleaner Production. 2016;133:358–367. 10.1016/j.jclepro.2016.05.123

[41] Kauppi H, Saikkonen P. Predicting US recessions with dynamic binary response models. The Review of Economics and Statistics. 2008;90(4):777–791. 10.1162/rest.90.4.777

[42] Liu W, Moench E. What predicts US recessions? International Journal of Forecasting. 2016;32(4):1138–1150. 10.1016/j.ijforecast.2016.02.007

[43] Devroye L. Nonuniform random variate generation. Handbooks in operations research and management science. 2006;13:83–121. 10.1016/S0927-0507(06)13004-2

[44] Johnson NL, Kemp AW, Kotz S. Univariate discrete distributions. vol. 444 John Wiley & Sons; 2005.

[45] Wright I. The duration of recessions follows an exponential not a power law. Physica A: Statistical Mechanics and its Applications. 2005;345(3):608–610. 10.1016/S0378-4371(04)01057-X

[46] de Bondt G, Vermeulen P. Business cycle duration dependence and foreign recessions. ECB Working Paper Series. 2018; (2205).

[47] FRED FRBoSL. OECD based Recession Indicators for Euro Area from the Period following the Peak through the Trough [EUROREC]; 2017. https://fred.stlouisfed.org/series/EUROREC.

[48] BEA. National Economic Accounts—Gross Domestic Product (GDP); 2017. https://www.bea.gov/national/index.htm#gdp.

