# Supplementary material for: Assessing the time intervals between economic recessions
Source: PLoS One. 2020 May 7;15(5):e0232615. doi: 10.1371/journal.pone.0232615 (PMC7205267; doi:10.1371/journal.pone.0232615)
Supplement: S1 Dataset — (PDF) [file pone.0232615.s001.pdf]

S1 Proof of Proposition 1

(i) For each $i = 1, \dots, n$, we use (21) and obtain

$$\begin{aligned}
\mathbb{P}[W_i \le w_i \,\Big|\, V_i; \alpha, \beta] &= \mathbb{P}\left[\exp\left(\frac{X_i + V_{i-1} - \alpha}{\beta}\right) - \exp\left(\frac{V_{i-1} - \alpha}{\beta}\right) \le w_i \,\Big|\, V_i; \alpha, \beta\right] \\
&= \mathbb{P}\left[\exp\left(\frac{X_i + V_{i-1} - \alpha}{\beta}\right) \le w_i + \exp\left(\frac{V_{i-1} - \alpha}{\beta}\right) \,\Big|\, V_i; \alpha, \beta\right] \\
&= \mathbb{P}\left[X_i \le \beta \log\left(w_i + \exp\left(\frac{V_{i-1} - \alpha}{\beta}\right)\right) + \alpha - V_{i-1} \,\Big|\, V_i; \alpha, \beta\right] \\
&= 1 - \exp\left(\exp\left(\frac{V_{i-1} - \alpha}{\beta}\right)\right. \\
&\quad \left. - \exp\left(\frac{\beta \log\left(w_i + \exp\left(\frac{V_{i-1}-\alpha}{\beta}\right)\right) + \alpha - V_{i-1} - \alpha + V_{i-1}}{\beta}\right)\right) \\
&= 1 - \exp(-w_i)\,.
\end{aligned}$$

Therefore, we have $W_i \sim \operatorname{Exp}(1)$, which concludes the proof of the first claim.

(ii) We verify that the joint distribution for X_1 and X_2 is given by

$$\begin{aligned}
\mathbb{P}[X_1 \le x_1, X_2 \le x_2] &= \mathbb{P}[X_1 \le x_1] \cdot \mathbb{P}[X_2 \le x_2 \,|\, X_1 \le x_1] \\
&= \mathbb{P}[X_1 \le x_1] \cdot \mathbb{P}[X_2 \le x_2 + q x_1]\,.
\end{aligned}$$

Consequently, the joint distribution of $X_1, \dots, X_n$ with $n > 2$ can be written as

$$\mathbb{P}[X_1 \le x_1, \dots, X_n \le x_n] = \prod_{j=1}^{n} \mathbb{P}\Big[X_j \le x_j + V_{j-1}\Big]\,,$$

and the density function for $\mathbf{X} = (X_1, \dots, X_n)$ is given by

$$f_{\mathbf{X}}(x_1, \dots, x_n \,|\, v_{i-1}; \alpha, \beta) = \prod_{i=1}^{n} f_{X_i}(x_i \,|\, v_{i-1}; \alpha, \beta)\,. \tag{23}$$

In order to show that the variables $W_1, W_2, \cdots, W_n$ are independent, we need to show that

$$f_{W_1,\dots,W_n}\,(w_1, \dots, w_n \,|\, v_{i-1}; \alpha, \beta) \;=\; \prod_{i=1}^{n} f_{W_i}\,(w_i \,|\, v_{i-1}; \alpha, \beta)\,. \tag{24}$$

Using the change of variables

$$W_i = \exp\left[\frac{X_i + V_{i-1} - \alpha}{\beta}\right] - \exp\left[\frac{V_{i-1} - \alpha}{\beta}\right],$$

and solving to $X_i + V_{i-1}$, we obtain

$$X_i + V_{i-1} = \beta \log\left(W_i + \exp\left(\frac{V_{i-1} - \alpha}{\beta}\right)\right) + \alpha.$$

The joint density of the $W_1, W_2, \cdots, W_n$, is given by

$$f_{W_1,\ldots,W_n}(w_1,\cdots,w_n \,|\, v_{i-1},\alpha,\beta) = \\ f\left(\varphi_1(x_1+v_0,\ldots,x_n+v_{n-1}),\cdots,\varphi_n(x_1+v_0,\ldots,x_n+v_{n-1}) \,|\, \alpha,\beta,q\right) \times J\,,$$

where

$$\varphi(x_1+v_0,\ldots,x_n+v_{n-1}) = \beta\log\left(w_1+\exp\left(\frac{v_0-\alpha}{\beta}\right)\right)+\alpha$$

and J is the determinant of the $(n\times n)$-matrix given by

$$\begin{aligned} J &= \det\left(\frac{\partial\varphi_i(x_1,\ldots,x_n)}{\partial w_j}\right)_{i,j} \\ &= \det\begin{bmatrix} \frac{\beta}{w_1+\exp\left(\frac{v_0-\alpha}{\beta}\right)} & 0 & \cdots & 0 \\ 0 & \frac{\beta}{w_2+\exp\left(\frac{v_1-\alpha}{\beta}\right)} & \cdots & 0 \\ \vdots & \vdots & \ddots & \vdots \\ 0 & 0 & \cdots & \frac{\beta}{w_n+\exp\left(\frac{v_{n-1}-\alpha}{\beta}\right)} \end{bmatrix} \\ &= \prod_{i=1}^{n}\frac{\beta}{w_i+\exp\left(\frac{v_{i-1}-\alpha}{\beta}\right)} \end{aligned} \tag{25}$$

Thus, according to (25), we have:

$$f_{W_1,\ldots,W_n}(w_1,\cdots,w_n \,|\, v_{i-1};\alpha,\beta) = \\ \left\{\prod_{j=1}^{n}\frac{1}{\beta}\exp\left[\frac{x_j+v_{j-1}-\alpha}{\beta}\overbrace{-\exp\left[\frac{x_j+v_{j-1}-\alpha}{\beta}\right]+\exp\left[\frac{v_{j-1}-\alpha}{\beta}\right]}^{-w_j}\right]\right\} \\ \times\prod_{i=1}^{n}\frac{\beta}{w_i+\exp\left[\frac{v_{n-1}-\alpha}{\beta}\right]}\,.$$

Replacing $x_i + v_{i-1}$ in the last equality, we obtain:

$$
\begin{aligned}
& f_{W_1,\ldots,W_n}(w_1,\cdots,w_n \,|\, v_{i-1};\alpha,\beta) \\
&= \frac{1}{\beta^n}\left\{\prod_{j=1}^{n}\exp\left(\frac{\beta\log(w_j+\exp((v_{j-1}-\alpha)/\beta))+\alpha-\alpha}{\beta}-w_j\right)\right\} \\
&\quad \times\ \beta^n \prod_{i=1}^{n}\frac{1}{w_i+\exp\left(\frac{v_{n-1}-\alpha}{\beta}\right)} \\
&= \prod_{j=1}^{n}\left(w_j+\exp\left(\frac{v_{j-1}-\alpha}{\beta}\right)\right)\times\exp(-w_j)\times\prod_{i=1}^{n}\frac{1}{w_i+\exp\left(\frac{v_{i-1}-\alpha}{\beta}\right)} \\
&= \prod_{j=1}^{n}\exp(-w_j)
\end{aligned}
$$

The last equality is independent of v_i, and hence $W_1, W_2, \cdots, W_n$ are independent and identically distributed, which concludes the proof of the second claim.
