# Supplementary material for: Assessing the time intervals between economic recessions
Source: PLoS One. 2020 May 7;15(5):e0232615. doi: 10.1371/journal.pone.0232615 (PMC7205267; doi:10.1371/journal.pone.0232615)
Supplement: S1 Table — National Bureau of Economic Research—NBER data. (PDF) [file pone.0232615.s002.pdf]

Table 3. U.S. Recessions. National Bureau of Economic Research - NBER data.

#	Peak month	Through month	#	Peak month	Through month
0	***	December 1854	17	January 1920	July 1921
1	June 1857	December 1858	18	May 1923	July 1924
2	October 1860	June 1861	19	October 1926	November 1927
3	April 1865	December 1867	20	August 1929	March 1933
4	June 1869	December 1870	21	May 1937	June 1938
5	October 1873	March 1879	22	February 1945	October 1945
6	March 1882	May 1885	23	November 1948	October 1949
7	March 1887	April 1888	24	July 1953	May 1954
8	July 1890	May 1891	25	August 1957	April 1958
9	January 1893	June 1894	26	April 1960	February 1961
10	December 1895	June 1897	27	December 1969	November 1970
11	June 1899	December 1900	28	November 1973	March 1975
12	September 1902	August 1904	29	January 1980	July 1980
13	May 1907	June 1908	30	July 1981	November 1982
14	January 1910	January 1912	31	July 1990	March 1991
15	January 1913	December 1914	32	March 2001	November 2001
16	August 1918	March 1919	33	December 2007	June 2009
