# Supplementary material for: Assessing the time intervals between economic recessions
Source: PLoS One. 2020 May 7;15(5):e0232615. doi: 10.1371/journal.pone.0232615 (PMC7205267; doi:10.1371/journal.pone.0232615)
Supplement: S2 Table — (PDF) [file pone.0232615.s003.pdf]

Table 4. European recessions [47].

i	Peak month	Through month	i	Peak month	Through month
0	***	01/03/1960	7	01/03/1991	01/09/1993
1	01/12/1963	01/05/1968	8	01/05/1995	01/02/1997
2	01/09/1970	01/04/1972	9	01/02/1998	01/02/1999
3	01/02/1974	01/07/1975	10	01/02/2001	01/08/2003
4	01/01/1977	01/10/1977	11	01/03/2008	01/07/2009
5	01/02/1980	01/01/1983	12	01/06/2011	01/04/2013
6	01/10/1985	01/04/1987			
