# Supplementary material for: Assessing the time intervals between economic recessions
Source: PLoS One. 2020 May 7;15(5):e0232615. doi: 10.1371/journal.pone.0232615 (PMC7205267; doi:10.1371/journal.pone.0232615)
Supplement: S3 Table — (PDF) [file pone.0232615.s004.pdf]

Table 5. Depth of U.S. recessions [48].

#	Peak month	Through month	Depth of recession
23	November 1948	October 1949	−5.3%
24	July 1953	May 1954	−5.9%
25	August 1957	April 1958	−10.0%
26	April 1960	February 1961	−4.8%
27	December 1969	November 1970	−4.0%
28	November 1973	March 1975	−4.7%
29	January 1980	July 1980	−7.9%
30	July 1981	November 1982	−6.5%
31	July 1990	March 1991	−3.4%
32	March 2001	November 2001	−1.3%
33	December 2007	June 2009	−8.2%
